# P-537. PAIRED - PAtIent Reported Experiences and perceiveD benefit of treatment with dolutegravir/lamivudine (DTG/3TC): a diverse sample of people with HIV-1 reporting high treatment satisfaction, good adherence, and high quality of life

**DOI:** 10.1093/ofid/ofae631.736

**Published:** 2025-01-29

**Authors:** Jihad Slim, Andrew P Brogan, Gavin Harper, Katie L Mycock, Abigail McMillan, Deanna Merrill, Gustavo Verdier

**Affiliations:** Saint Michael’s Medical Center, Newark, NJ, USA, Newark, New Jersey; ViiV Healthcare, San Diego, California; Adelphi Real World, Bollington, England, United Kingdom; Adelphi Real World, Bollington, England, United Kingdom; Adelphi Real World, Bollington, United Kingdom, Bollington, England, United Kingdom; ViiV Healthcare, San Diego, California; ViiV Healthcare, Montréal, QC, Canada, Pointe-Claire, Quebec, Canada

## Abstract

**Background:**

Understanding the real-world experiences of people with HIV-1 (PWH) is essential to tailoring HIV treatment to PWH needs. PAtIent Reported Experiences and perceiveD benefit of treatment with dolutegravir/lamivudine (DTG/3TC) (PAIRED) examined PWH experiences in the United States.

**Figure:**
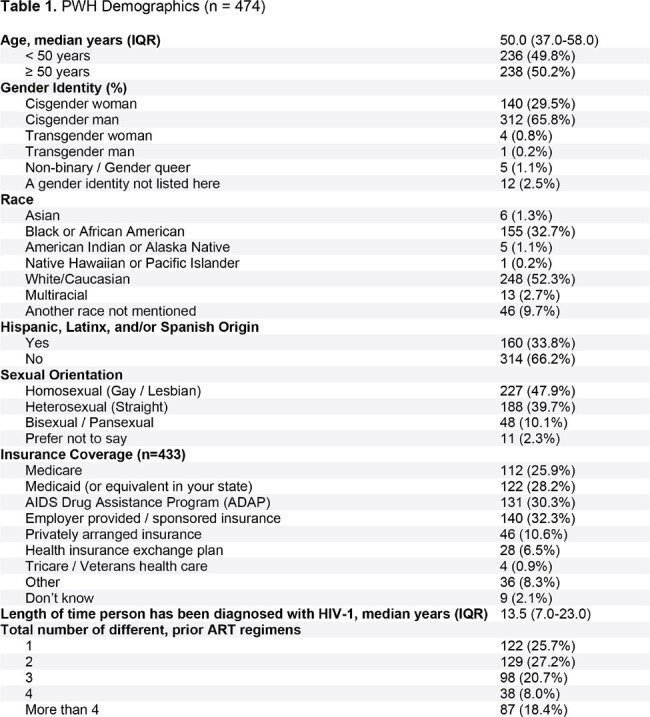

**Methods:**

PAIRED comprised a cross-sectional survey and in-depth qualitative interviews of stable-switch PWH ≥ 18 years, receiving DTG/3TC for ≥ 3 months. A mixed recruitment methodology (site-led and community outreach) was employed, and the survey included validated instruments [HIV-Treatment Satisfaction Questionnaire (HIV-TSQs), Adelphi Adherence Questionnaire™ (ADAQ), PoZQoL]. The survey results presented here are descriptive.

**Figure:**
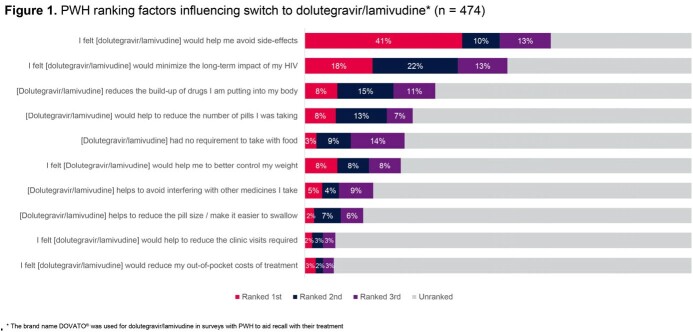

**Results:**

PAIRED represented a diverse sample of 474 participants (31% female sex at birth, 48% non-White, and 50% ≥ 50 years) (Table 1). Median time since HIV diagnosis was 13.5 years (IQR: 7.0-23.0) and most (74%) had taken ≥ 2 previous antiretroviral (ART) regimens. The majority switched from either bictegravir/emtricitabine/tenofovir alafenamide (28%) or abacavir/dolutegravir/lamivudine (28%) to DTG/3TC, and 73% had taken their immediate previous treatment for > 12 months. Majority of participants (62%) had taken DTG/3TC for > 12 months. When asked to rank factors influencing switch to DTG/3TC, PWH reported avoidance of side effects and minimizing long-term impact as top 2 factors (Figure 1). Using the HIV-TSQs, PWH reported high satisfaction with dolutegravir/lamivudine. Out of a maximum score of 60, the median total HIV-TSQs score was 57.0 (IQR: 52.0-60.0). Out of a maximum score of 65, participants had an overall median PozQoL score of 47.0 (IQR: 38.5-55.0), indicative of high quality of life. PWH reported improved treatment satisfaction with DTG/3TC compared with their previous ART regimen (68% vs 31% very satisfied) (Figure 2) and reducing the number of medicines was extremely or very important to 79% of PWH (Figure 3). Good adherence was observed using the ADAQ [median ADAQ^©^ score 0.4 (IQR: 0.2-0.5); possible scores 0-4], with 89% of PWH reporting never or rarely missing a DTG/3TC dose.

**Figure:**
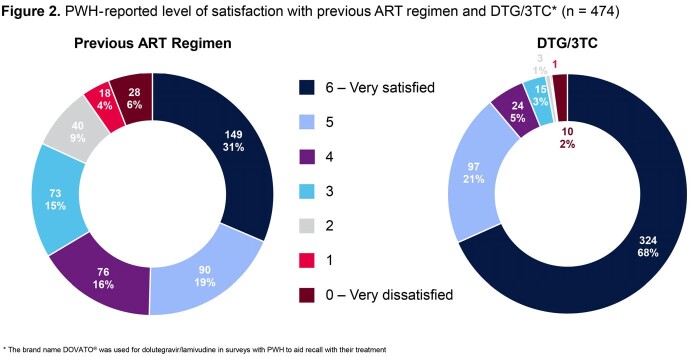

**Conclusion:**

PAIRED represented a diverse sample of PWH switching to DTG/3TC who were highly satisfied with treatment resulting in good adherence and high quality of life.

**Figure:**
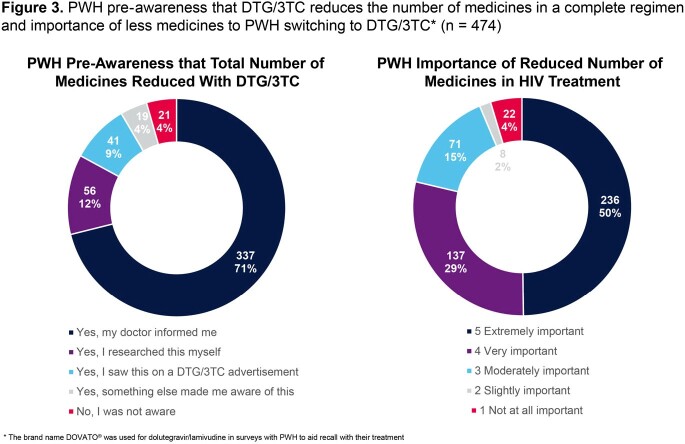

**Disclosures:**

**Jihad Slim, MD, FACP**, AbbVie: Grant/Research Support|AbbVie: Honoraria|AbbVie: Speaker Bureau|Gilead Sciences, Inc.: Grant/Research Support|Gilead Sciences, Inc.: Honoraria|Gilead Sciences, Inc.: Speaker Bureau|Merck: Grant/Research Support|Merck: Honoraria|Merck: Speaker Bureau|Theratechnologies: Advisor/Consultant|Theratechnologies: Honoraria|ViiV Healthcare: Advisor/Consultant|ViiV Healthcare: Grant/Research Support|ViiV Healthcare: Speaker Bureau **Andrew P. Brogan, PhD**, GSK: Stocks/Bonds (Public Company)|ViiV Healthcare: Employee **Gavin Harper, BA**, Adelphi Real World: Employee|ViiV Healthcare: Contract for this analysis **Katie L. Mycock, MChem**, Adelphi Real World: employee|ViiV Healthcare: Contract for this analysis **Abigail McMillan, MSc**, Adelphi Real World: employee|ViiV Healthcare: Contract for this analysis **Deanna Merrill, PharmD, MBA, AAHIVP**, GSK: Stocks/Bonds (Public Company)|ViiV Healthcare: Employee **Gustavo Verdier, BSc, BPharm, MBA**, ViiV Healthcare: Employee

